# Mapping and Discriminating Rural Settlements Using Gaofen-2 Images and a Fully Convolutional Network

**DOI:** 10.3390/s20216062

**Published:** 2020-10-25

**Authors:** Ziran Ye, Bo Si, Yue Lin, Qiming Zheng, Ran Zhou, Lu Huang, Ke Wang

**Affiliations:** 1 Institute of Applied Remote Sensing and Information Technology, College of Environmental and Resource Sciences, Zhejiang University, Hangzhou 310058, China; smd_ye@zju.edu.cn (Z.Y.); bo_si@zju.edu.cn (B.S.); joyelin_2018@zju.edu.cn (Y.L.); qmzheng@zju.edu.cn (Q.Z.); 3160100350@zju.edu.cn (R.Z.); kwang@zju.edu.cn (K.W.); 2 Department of Biological Sciences, National University of Singapore, 14 Science Drive 4, Singapore 117543, Singapore; 3 The Rural Development Academy, Zhejiang University, Hangzhou 310058, China

**Keywords:** rural settlements, fully convolutional network, multi-scale context, high spatial resolution images

## Abstract

New ongoing rural construction has resulted in an extensive mixture of new settlements with old ones in the rural areas of China. Understanding the spatial characteristic of these rural settlements is of crucial importance as it provides essential information for land management and decision-making. Despite a great advance in High Spatial Resolution (HSR) satellite images and deep learning techniques, it remains a challenging task for mapping rural settlements accurately because of their irregular morphology and distribution pattern. In this study, we proposed a novel framework to map rural settlements by leveraging the merits of Gaofen-2 HSR images and representation learning of deep learning. We combined a dilated residual convolutional network (Dilated-ResNet) and a multi-scale context subnetwork into an end-to-end architecture in order to learn high resolution feature representations from HSR images and to aggregate and refine the multi-scale features extracted by the aforementioned network. Our experiment in Tongxiang city showed that the proposed framework effectively mapped and discriminated rural settlements with an overall accuracy of 98% and Kappa coefficient of 85%, achieving comparable and improved performance compared to other existing methods. Our results bring tangible benefits to support other convolutional neural network (CNN)-based methods in accurate and timely rural settlement mapping, particularly when up-to-date ground truth is absent. The proposed method does not only offer an effective way to extract rural settlement from HSR images but open a new opportunity to obtain spatial-explicit understanding of rural settlements.

## 1. Introduction

Since the reform and opening-up, drastic urbanization has been taking place in China. In a stark contrast, the development of rural areas, however, is not in concert with that of urban areas, but is greatly lagging behind and restricted. Mass population migration, from rural to urban areas, has given rise to a succession of impacts on rural areas, including population decline, industry recession and land abandonment [1,2]. In 2018, China stepped up its efforts to revitalize rural regions. Building the new style of rural community with better infrastructure is one of the important measures to improve the wellbeing of rural people. Thus, a spatial-explicit understanding of rural settlements regarding their distributions is of critical essence to effective land management and policy making. 

Satellite-based earth observation is a key enabler for capturing spatial information of buildings in rural areas. High spatial resolution (HSR) images open new opportunities for slums and informal settlement detection and rural land cover mapping [3,4]. Compared with medium resolution image which mainly offers spectral information (in terms of a single image) [5], using HSR images can leverage both spectral and spatial information. HSR image analysis basically relies on image classification (e.g., pixel-based) and segmentation (e.g., Object-Based Image Analysis (OBIA)) techniques [6,7], with the help of handcrafted features extracted from spectral (e.g., reflectance and spectral indices, like Normalized Difference Vegetation Index (NDVI)) and spatial (texture statistics, morphological profiles, and oriented gradients) [8,9]. With an ever-increasing focus on rural areas, satellite images have been extensively used for rural settlement mapping [10,11]. Nevertheless, applying HSR images to rural settlement detection remains a challenging task due to the following issues. First, the size and spatial distribution of rural settlements varies significantly, e.g., clustered or scattered, because rural planning is changing over time. Second, the intra-class variation makes it difficult to distinguish rural settlements from construction materials when using spectral information alone. Third, when considering large spatial areas, the spectral and spatial responses from ground objects present an extremely complex pattern [8]. In order to discriminate rural settlements, more context information is required in the classification. In previous studies, such as [12,13], landscape metrics were used as the spatial contextual information to identify rural settlements from HSR satellite imagery. These methods exploit tailored segment-based features and have achieved acceptable performance. However, parameters optimization and handcrafted features selection are laborious tasks and are highly hinged upon expert experience, and trial-and-error tests. 

Deep learning methods, such as convolutional neural networks (CNNs), have shown great potential for automatically features learning without human intervention. CNNs are able to generate robust feature representations hierarchically and have become increasingly popular in image classification and semantic segmentation [14]. Semantic segmentation for remote sensing data usually refers to extracting terrestrial objects from earth observation images using CNNs model, that is, each pixel is assigned a semantic label in pixel-based classification [15]. The fully convolutional network (FCN) [16] extends CNNs to segmentation, emerging as the preferred scheme for semantic labeling tasks. FCN inputs images of arbitrary sizes into a standard CNN, extract feature maps using layer-wise activation and abstraction, and then output high resolution predictions in an end-to-end fashion. The essential advantages of FCNs are the intrinsic ability to enhance feature representation and the flexibility to accept input images of any size. Previous studies have applied FCN and its variants to detect buildings and settlements [17,18,19]. It is further found that incorporating contextual relations in CNNs can improve classification accuracy [20,21]. Nevertheless, most of the above-mentioned approaches are designed to extract target objects in urban areas from the standard datasets [22]. In rural areas, built-up areas tend to be sparse and can be easily omitted [23]. Due to the significant differences in the appearance of urban and rural buildings, directly employing existing deep approaches to map rural settlements does not guarantee good performance. In addition, the difficulty in image interpretation increases sharply as the spatial resolution increases. Therefore, we wish to make use of the advantages of deep learning technique to contribute to the area of rural settlements identification in HSR images. By far, only a few studies applied FCNs to extract rural residential areas [24,25], and most of them were limited by the spatial resolution of images or the extent of application. The effectiveness of FCNs in rural settlement mapping using HSR images requires further in-depth examination. In short, it is imperative to develop an effective method to buttress automatic extraction of rural settlements using HSR images.

The overall objective of this paper is to develop a framework for automatically identifying rural settlements in HSR satellite images based on deep learning technique. Our main contributions are: (1) This application introduces a deep FCN method to recognize rural settlements. Specifically, dilated convolutions are used to extract deep features at high spatial resolution. (2) A multiple scale context subnetwork, which adopts a popular squeeze and excitation (SE) module [26] to aggregate multi-scale context, is exploited to generate discriminative representations. The proposed deep learning-based rural settlement extraction scheme can flexibly take multi-spectral HSR images as input to distinguish different types of rural settlements.

## 2. Study Area and Data

**Figure 1:**
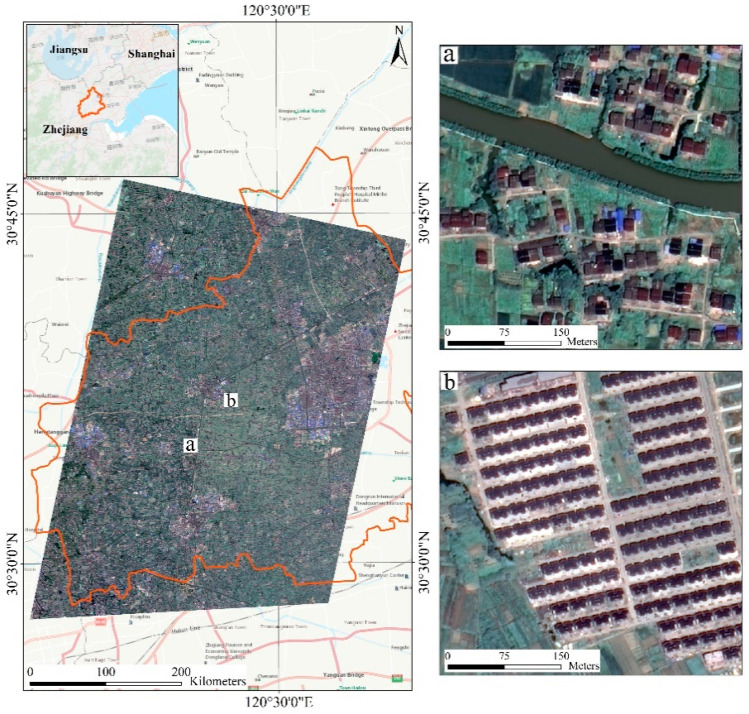
GaoFen-2 image of Tongxiang study area on July 2016. Example of (**a**) low-density rural settlement and (**b**) high-density rural settlement.

In this research, eleven towns of Tongxiang County were selected as study area, a typical rural region undergoing rapid rural development and transformation in the Yangtze River delta of China (120°30′13″E, 30°41′10″W). Tongxiang, located in the Hangjiahu plain, has a temperate climate with distinct seasonality. Since 2000, several land consolidation projects have been carried out to promote the construction of new countryside. Currently, the construction and renovation of countryside are still ongoing in Tongxiang, so the old scattered low-rise houses are mixed with uniformly planned residential buildings. Therefore, this area is an ideal study area to examine our proposed method. We preliminarily divide these settlements into two categories. Figure 1 shows examples of two types of rural settlements in the study area—low-density settlements and high-density settlements.

1. Low-density settlements (LDS): most of LDS are old-style rural settlements which are scattered and disorderly distributed and have different orientations. These low-density rural settlements are mainly located close to rivers and streams in support of farming and transportation of smallholders. The boundaries of low-density settlements are obscured by the surrounding vegetation.
2. High-density settlements (HDS): newly built residential areas where multi-story buildings accommodate several families. Such settlements have a higher building density than low-density settlements, and buildings inside these settlements have an identical spacing and the same surface. High-density settlements mainly distribute adjacent to the newly built transportation roads, providing easy access to nearby towns.

China’s GaoFen-2 (GF-2) HSR images were used, comprising four multispectral bands (MSS) with a spatial resolution of 4 m and a panchromatic band (PAN) with a spatial resolution of 1 m. The acquisition time of two images was on July 2016. And we collected the land use data of the study area in 2015 (provided by the Bureau of Land and Resources, Tongxiang, China) to generate ground truth data.

## 3. Methods

**Figure 2:**
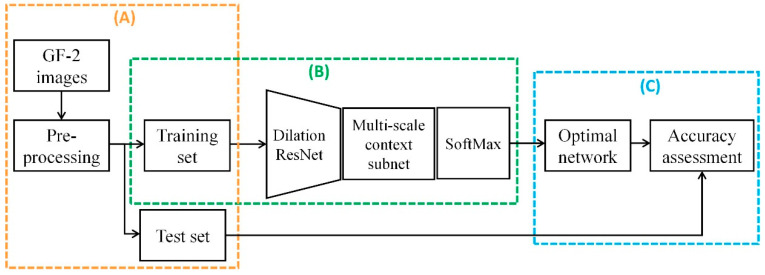
Flowchart of the proposed research framework: (**A**) generate data sets, (**B**) model training, and (**C**) accuracy assessment.

Figure 2 demonstrates the flowchart of our proposed method. First, the GF-2 image was pre-processed and split into training set and test set. Second, the trained model was used to classify the rural settlements. Finally, accuracy assessment was conduct on the test set. The details of the proposed method are described in the following subsections.

### 3.1. Data Preprocessing

For the cloud-free and haze-free atmospheric condition in the acquired image, there was no need for atmospheric correction in the preprocessing. After orthorectification, the MSS image and PAN images were then fused using the Gram–Schmidt pan-sharpening method [27]. The fusion image (1 m) had a dimension of 29,970 × 34,897 pixels, equivalent to about 700 km^2^. The reference map was generated based on (1) the land-use change surveying map and (2) visual interpretation by local experts. Note that the ground truth data in our study was spatially sparse, which thereby was more in line with real-world scenarios, where densely annotated data is rarely available.

### 3.2. Rural Settlement Detection Using FCN

#### 3.2.1. Dilated Residual Convolutional Network

The task of automatically extracting settlement information in a large rural region can be formulated as a semantic labeling problem to distinguish pixels of categories. In this section, we wish to put forward an end-to-end method based on semantic segmentation scheme to identify rural settlements. Our approach used ResNet50 architecture as the feature extractor of FCN-based method. The ResNet consists of five stages of convolutional layers. In the first stage, a convolution layer performs 7 × 7 convolution and is followed by a maxpooling operation, which outputs the features that are a quarter of the size of the original image. In the remaining four stages, each stage contains several blocks, which is a stack of two 3 × 3 convolutional layers. Moreover, two types of shortcut connection are introduced in the blocks to fuse input feature maps with output feature maps according to the size of input and output features. More details about ResNet50 can be found in [28].

As the network goes deeper, the resolution of feature maps becomes smaller while the channels increase. For example, the output feature maps of the last stage are reduced to 1/32 of the size of the original input. Compared with the complex background in HSR images, the objects of our interest (i.e., rural settlements) are smaller and sparser. Besides, the loss of spatial information due to the progressive down-sampling in the network is harmful for identifying small objects. To retain the large receptive field and increase spatial resolution in higher layers of network simultaneously, we adopt convolutions with dilated kernels into the ResNet. In the last two stages of original ResNet50, the strided convolution layer, which is used to reduce the output resolution at the beginning of each stage, is substituted by a convolution layer with the stride of 1 (meaning no downsampling). Recent studies [29,30] indicate this conversion does not affect the receptive field of the first layer of the stage, but it reduces the receptive field of subsequent layers by half. In order to increase the receptive field of those layers, convolutions with different dilation factors were adopted. Specifically, the dilation ratio of convolutional kernel was set as 2 and 5 in the fourth and the fifth stage, respectively. Dilated convolutions were thus expected to enlarge the receptive field of layers and to generate features with high spatial resolution. As a result, the output size would increase from 1/32 to 1/8 of the input image. 

#### 3.2.2. Multi-Scale Context Subnetwork

**Figure 3:**
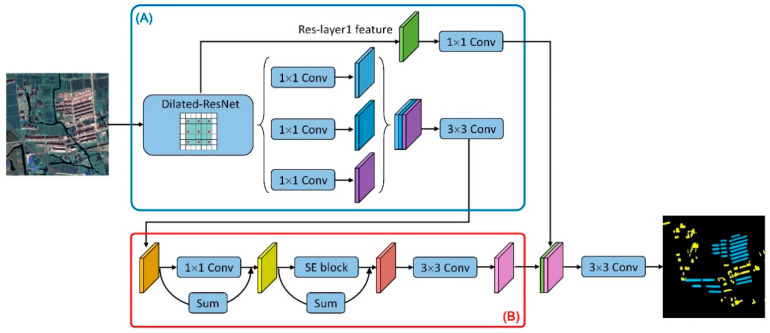
Overview of the proposed detection architecture. (**A**) the Dilated-ResNet extracted multi-level features with high spatial resolution; (**B**) the context subnetwork exploited the multi-scale context and mapped features to desired outputs.

Some upgraded low-density houses and high-density buildings may have used similar roofing materials. In order to distinguish between the two categories of rural settlements, their spatial distribution and context need to be fully considered. Due to a great variety of the size of rural settlements, it is necessary to capture multiple scales information to identify objects in rural residential areas. Instead of using multiple rescaled versions of an image as input to obtain multi-scale context information, we introduced a multi-scale spatial context structure to handle the scale variation of rural residential objects. Commonly, the deep layers in CNNs respond to global context information and the shallow layers are more likely to be activated by local texture and patterns. Benefit from the dilation convolution maintaining spatial resolution, the three scale-levels features extracted by the backbone ResNet50 can be utilized at the same time. Our structure further enhanced the information propagation across layers. As shown in Figure 3, the output features of last three stages were filtered by 1 × 1 convolution layers to shrink the channel to 256 and then concatenated together. It is notable that we appended 3 × 3 convolution on the merged map to generate the subsequent feature map, which was to reduce the misalignment when fusing features of different levels. Secondly, a residual correction was introduced to alleviate the lack of information during feature fusion. Finally, feature selection was conducted by employing an advanced channel encoding module named “squeeze and excitation” block (SE block) [26], which adaptively recalibrated channel-wise feature responses. Once features were input into the module, global average pooling was used to generate a vector as channel-wise descriptors of the input features. Subsequently, two fully connected layers were applied to the vector to learn nonlinear interaction among channels. The sigmoid activation function would then generate a weight vector as a scale factor for the class-dependent features. The features refined by the above reweighting process had discriminative representations, which were helpful for object identification. Based on abundant positioning and identification information, the successive 3 × 3 convolution layer was expected to produce more accurate features. Finally, the refined deep features were then concatenated with the corresponding low-level features (e.g., Res-layer1 in ResNet50) in order to restore spatial details. After the fusion, we applied another 3 × 3 convolution layer and a simple bilinear upsampling to get the final segmentation. Table S1 shows the specific design of our segmentation network.

#### 3.2.3. Multi-Spectral Images-Based Transfer Learning

CNNs are generally data-driven approach and are usually trained on large datasets. In practice, a sufficiently large data set is rare. Instead, it is more practical to use a deep network previously trained on a big dataset (e.g., ImageNet) as an initial model or a feature extractor for the target task. This scheme is known as transfer learning [31]. In brief, the idea of transfer learning is to leverage knowledge from the source domain to boost learning in the target domain, as features of CNNs are more generic in early layers. Compared with training from scratch, the cost of fine-tuning the pre-trained network is much lower. Several attempts have been made to improve the learning task in remote sensing datasets by using transfer learning [32,33,34].

The ResNet50 is initially designed for RGB images [28]. To better adapt to multispectral remote sensing data which have the red (R), green (G), blue (B) and near-infrared (NIR) bands, the network was expanded to take advantage of more input bands than RGB. Different from the idea of using an additional convolution layer at the beginning of network [35] or adding a branch to accept multiband inputs [34], we directly modified the original 7 × 7 convolution layer in the first stage of ResNet to make it flexible to receive multispectral images and output 64 features.

### 3.3. Method Implementation and Accuracy Assessment

A total of 7605 tiles of a size of 256 × 256 pixels were cropped from the training area of the preprocessed GF-2 imagery, and we randomly selected about 20% of image patches as the validation set. Data augmentations consisting of flipping and rotation of 90 degrees were applied to enlarge the training set. The proposed network was trained on a 24 GB Nvidia P6000 GPU. The weights of network were initialized using the pre-trained ResNet50 model. We copied the weights of the first channel to initialize the newly added channel in the first convolution layer. An adaptive algorithm Adam [36] was employed as the optimizer, and the learning rate was set to 0.001. A batch size of 8 was used, running the optimizer for 30 epochs with an early stopping strategy which stopped training process when the monitored quantity (i.e., validation loss) had stopped improving for 5 epochs. The proposed method was implemented on the Pytorch framework.

**Figure 4:**
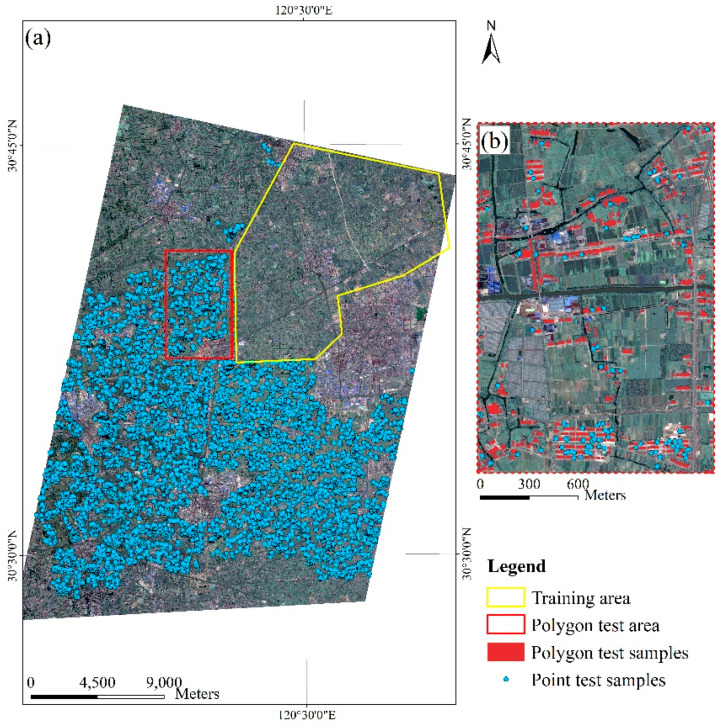
The (**a**) Tongxiang data set used in the experiments. (**b**) Example of test samples.

Figure 4 shows the training area and the test area in the experiment. The point test samples were all over the entire study area except the training area. In order to further evaluate the area accuracy, we selected a small area in the test area as the polygon test subset, and rural settlements in the polygon test subset were densely labeled. The random point generating algorithm in ArcGIS [37] was applied to generate a total of 11,628 sample points. After that, we manually annotated these sample points based on higher resolution images of Google Earth and visual interpretation. In addition to the two types of settlements about which we were concerned, all other objects in the image were included in the background category. Table 1 lists the number of test set samples.

Following the previous studies, the overall accuracy (OA), producer’s accuracy (PA), user’s accuracy (UA) and Kappa coefficient (Kappa) [38] were used to assess the performances of methods. The producer’s accuracy represents the probability that pixels of a category are correctly classified, whereas the user’s accuracy indicates the probability that the classified pixels belong to this category. Overall accuracy is the percentage of correctly classified pixels. The Kappa analysis is a discrete multivariate technique used in accuracy assessment to test whether one error matrix is significantly different from another [39], and Kappa coefficient calculated based on the individual error matrices can be regarded as another measure of accuracy.

## 4. Results and Discussions

### 4.1. Rural Settlements Identification

**Figure 5:**
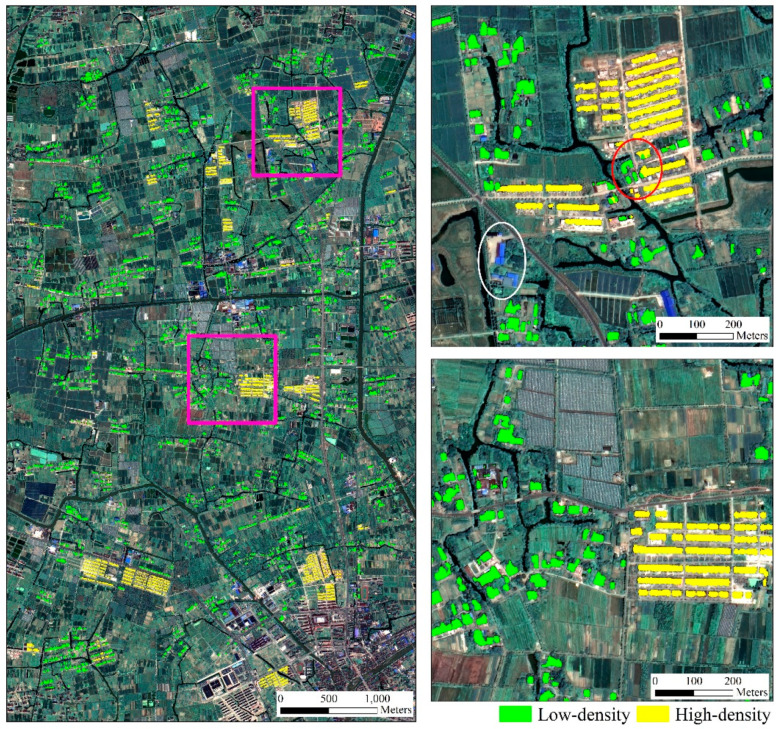
Classification result of the polygon test area.

Figure 5 shows the resulting rural settlements of our study area. Table 2 and Table 3 present confusion matrices on test sets. The proposed method achieved the OA of 98.31% with a Kappa coefficient of 0.9724 on the point test set, and the UA and PA of two settlements classes reached about 98%. The classification accuracy on polygon-based testing samples was different, the accuracies of low-density class (UA of 88.00% and PA of 84.97%) were higher than those of high-density class (UA of 85.22% and PA of 84.68%). In terms of overall classification, the Kappa coefficient of 0.8591 in the polygon-based testing method were lower than that of the point-based test set. This was because the polygon-based test method had strict requirements on the object boundary. Visual interpretation indicates that the proposed method can effectively distinguish rural residential areas from other man-made structures (white circle in Figure 5). It was observed that the footprints of HDS were more smoothed than the LDS, where the latter ones were inclined to be obscured by the surroundings, e.g., tress and shadows. The introduction of multi-scale context made it easier for HDS with relatively uniform scales to be detected, which was reflected by the PA. In addition, a few LDS houses on the edge of HDS were misclassified into isolated houses within HDS. This was caused by a similar roofs and ambient vegetation (red circle in Figure 5). It further suggests that the polygon-based testing method is necessary. Previous studies considered recognition accuracy, but sometimes did not include area accuracy of rural settlements.

### 4.2. Ablation Experiments of Model

**Figure 6:**
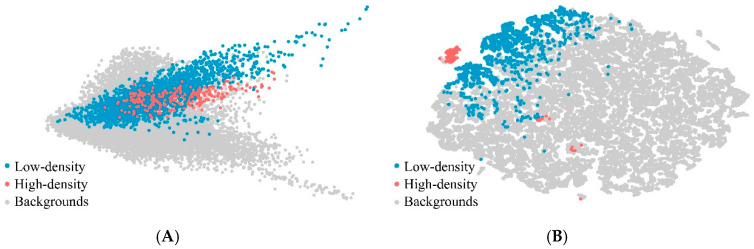
Visualization of test set samples before (**A**) and after recalibration (**B**) with SE block. Different colors represent different categories.

This study proposed a deep learning-based approach to extract rural settlements using HSR images. Experiments were carried out to explore the contribution of each part of the proposed deep method. Table 3 compares the performance of models with different settings based on the polygon test set. As showed in Table 4, when applying the original ResNet50 for segmentation, the accuracies of low-density class (UA of 82.50% and PA of 83.30%) were higher than those of high-density class (UA of 80.45% and PA of 67.75%). The low classification of PA indicates extracting HDS is rather challenging than LDS. When the last two stages of the baseline network were replaced by dilated convolutions, the PA index of high-density class was increased significantly by about 9%, while the UA of high-density class and the PA of low-density class had a moderate decrease. These indicated that the sub-module (+Dilation) was still insufficient. The possible reasons for the inconsistent changes in accuracies are the contradiction between the improvements brought by dilated convolutions and the defects of using single-scale feature. When comparing with the sub-module (+Dilation), another sub-module (+Dilation+Multiscale) yielded better accuracy on high-density class (UA of 84.88% and PA of 83.19%), with a slight increase in PA of low-density class, indicating that multi-scale context information enhanced the recognition power of the model. From Table 4, it can be seen that the proposed model achieved the largest OA of 98.68% with a Kappa coefficient of 0.8591. At the top of the aggregation layer, SE block captured feature dependencies in the channel dimension, and such feature selection process further improved the model performance. Figure 6 shows the visualization results of test set samples before and after recalibration with the SE block, implemented by t-SNE [40] technique. After the SE block, some samples of rural settlements classes gathered and were away from the background group, implying that the output of the channel relation module is more helpful for this classification task.

### 4.3. Data Input Strategies

**Figure 7:**
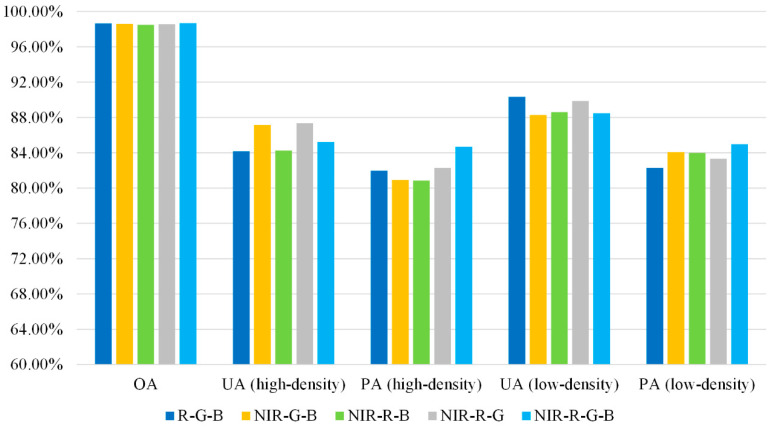
Accuracy assessment of different data input strategies.

Further experiments on two data input strategies, i.e., four channels and three channels, were conducted on the polygon test set. It was found that the classification accuracy of NIR-R-G-B composite images was slightly better than that of the R-G-B, but no significant difference was observed (Figure 7). It indicates that additional information of NIR band has positive effects on rural settlement extraction, while the powerful ability of CNNs to extract texture information from R-G-B images offset the gap between the two input strategies. Although the NIR band did not provide as great an improvement in accuracy as the DSM information [34], the strategy of using pre-trained weights of RGB data to initialize multispectral remote sensing images could be extended in the future.

### 4.4. Comparative Studies with Different Methods

Five state-of-the-art methods were compared, including an object-based image analysis (OBIA) method and four FCN based deep models. These methods have been proven effective in delineation of settlements and/or object detection for satellite images. The detailed information of each method can be found in the publication and we just briefly summarized their key technologies.

1. OBIA [12]: a novel object-based image classification method which integrates hierarchical multi-scale segmentation and landscape analysis. This method makes use of spatial contextual information and subdivides different types of rural settlements with high accuracy.
2. FCN [25]: a proposed fully convolutional network which comprises an encoder based on the VGG-16 network and a decoder consists of three stacked deconvolution layers. As far as we know, this is the first time that a deep learning FCN model has been used for rural residential areas extraction.
3. UNet [41]: a robust CNN architecture which consists of two symmetric contracting and expansive paths, which are made up of successive convolution layers. UNet is one of the deep learning methods often applied in the remote sensing field due to its efficiency and simplicity [42].
4. SegNet [43]: an encoder-decoder architecture uses the pooling indices to perform upsampling. It is a classic and efficient model that is often used as a baseline for semantic segmentation. Persello et al. [44] successfully delineated agricultural fields in smallholder farms from satellite images using SegNet.
5. DeeplabV3+ [20]: a state-of-the-art semantic segmentation model combining spatial pyramid pooling module and encode-decoder structure. It has achieve a performance of 89% on the PASCAL VOC 2012 semantic segmentation dataset.

**Figure 8:**
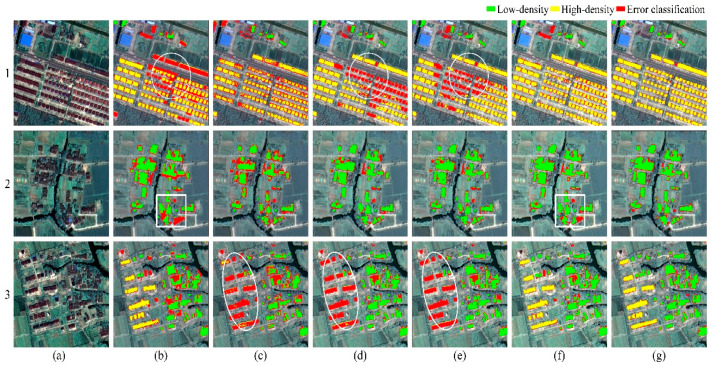
Example of results on Tongxiang polygon test set. (**a**) Original images, (**b**) OBIA, (**c**) FCN, (**d**) UNet, (**e**) SegNet, (**f**) DeeplabV3+, (**g**) The proposed method.

Figure 8 demonstrates samples selected from classification results of all six methods based on the polygon test set. Quantitative results are presented in Table 5. In terms of overall performance, all six methods exhibited a high accuracy (OA > 0.97), and the results of the Kappa coefficient were consistent with OA. However, there were obvious differences about class-specific measures among the methods. With regards to UA, PA, the proposed method achieved the best accuracies, slightly better than the accuracies of DeeplabV3+. The UA and PA of SegNet and UNet were relatively close, but not as good as the proposed method. Unfortunately, the PA of FCN was lowered than other methods, indicating FCN is not the best choice to distinguish settlements pixels. Finally, the results of OBIA indicate that, for high-density class, the object-based method performs better than SegNet and UNet in PA significantly and slightly worse in UA, but lags far behind in Kappa values.

For the low-density class, all deep techniques, except FCN, achieved satisfying performance because the number of low-density pixels was relatively large in the training data, which was an advantage for data-driven deep learning methods. The FCN model only used deep features for classification, and the loss of spatial information led to blurred building boundaries. In contrast, the object-based method performed better for HDS identification. Unlike the end-to-end deep methods, the performance of object-based method was heavily depended on the scale parameter of segmentation. The new-style HDS’ scale was relatively uniform and could be effectively extracted using OBIA method, even with a small sample size. Comparatively, LDS had a large size variation and were more sensitive to the choice of segmentation scale. Although the multi-context OBIA method exploited multiple segmentation scales to obtain the objects to be classified, it was still insufficient to separate the LDS of different sizes from the surrounding vegetation. Figure 8b shows that the OBIA method tends to intermingle the adjacent houses with vegetation or ground due to an improper segmentation scale selection. Moreover, manually designed features reduced the generalizability of methods in a large region. SegNet and UNet struggled in scenes where LDS and HDS are co-existed and mixed (Figure 8d,e). Compared with SegNet and UNet, using multi-scale context information helped the proposed method and DeeplabV3+ to reduce the misclassification of HDS. However, it inevitably induces some ambiguities on the boundaries of polygons (Figure 8f,g).

Table 6 lists the computing time of the proposed method and other methods. For the OBIA method, the segmentation and classification were conducted separately, and thereby showed the least time consumption. Instead, deep learning methods were end-to-end approaches. Among deep learning methods, FCN consumed fewer computing resources and had the shortest inference time because FCN had abandoned the full connection layers with lots of parameters. Therefore, the lack of feature representation capability limited the performance of FCN in this task. The proposed model showed similar model size and inference time with SegNet, but it took less training time to reach convergence. UNet and DeeplabV3+ have more parameters and they take longer to converge. Overall, the proposed method is more efficient.

### 4.5. Analysis and Potential Improvements

In our analysis, we found that all selected deep methods, except the proposed method and DeeplabV3+, were not as effective in the high-density category as in the low-density category. One possible reason was that the downsampling operation of the comparative methods was aggressive. Instead, using dilated residual convolutional network retained the spatial resolution of features. Given the input image patch (256 × 256), the deepest feature map of the proposed network maintains an appropriate size (32 × 32), which helps to restore the geometry of settlements. In this way, the accuracy of HDS increased greatly. However, the problem of scale selection remained. Unsynchronized scales of different types of settlements made it difficult to determine the optimal scale. The proposed multi-scale context subnetwork involved multiple scales, thereby reducing the dependence on a single optimal scale to a certain extent. However, the minimum scale (32 × 32) of representations applicable in the Tongxiang dataset may not match other HSR data. Thus, if the proposed method is applied to other data, determining an appropriate scale range would depend on the size of settlements objects and input images.

In some areas, HDS and LDS could not be easily recognized as they were in similar shapes, structures. Deep features at multiple scale could handle such complex patterns of settlements objects of different sizes, and the SE block modeled the global contextual relation of fused features, enabling feature selection in the channel dimension. The multi-scale context subnetwork gave more confident predictions at pixel level. The way that DeeplabV3+ uses the spatial pyramid module to encode multi-scale context information has achieved similar effects as our context subnetwork. The experimental results demonstrated that the proposed multi-scale network distinguish two types of settlements objects effectively. Nevertheless, contours of rural settlements needed to be further refined. Blurred object boundaries were an inherent and common defect of CNN-based semantic segmentation models. The downsampling process in the CNN model inevitably lost spatial details, which was detrimental to the preservation of edge information. However, this was a trade-off between spatial resolution and semantic feature representation of segmentation models. Our results showed that in this application, the use of dilated convolution instead of downsampling alleviated the loss of boundary details.

Segmentation and classification are conducted separately in OBIA method, which makes the classification result greatly affected by the performance of segmentation algorithm. Besides, handcrafted features used in OBIA are difficult to achieve an optimal balance between discriminability and robustness, since these features cannot easily consider the details of real data, especially in the case of HSR images that images can change a lot in large extent [45]. Instead, deep learning methods conduct segmentation and classification at the same time, and the classification results in Table 5 prove the superiority of the proposed method. Though deep learning methods take longer to train, it takes only a few seconds for a trained network to classify images. From the perspective of application, this is more applicable to the situation of big data of HSR images. Moreover, observation from the OBIA results, image segments could preserve the precise edges if under the appropriate segmentation scale. According to this observation, it is promising to combine the segmentation of OBIA and the feature representation of deep learning to classify rural settlements. Furthermore, this leaves open the question of whether a non-differentiable segmentation algorithm can be integrated into CNNs. In future, we hope to find a way to integrate the advantage of OBIA segmentation into the proposed framework of a deep network for rural settlement mapping.

## 5. Conclusions

Rural settlements classification using HSR remotely sensed image remains a challenging task, due to the intra-class spectral variation and spatial scale variation. This paper presents an effective rural settlements extraction method based on a deep fully convolutional network (FCN) from HSR satellite images. In the proposed multi-scale FCN model, dilated convolution was utilized to extract feature representations with high spatial resolution. A subnetwork improved the discrimination power of the network by aggregating and re-weighting multi-scale context information across layers. High spatial resolution representations and multi-scale context information helped to locate and further subdivide rural settlements. Experimental results on GF-2 images acquired over a typical rural area located in Tongxiang, China, showed the proposed method produced the most accurate classification results of rural settlements, comparing with other state-of-the-art methods and the sub-modules. In summary, our proposed method was promising in terms of its potential for rural settlements extraction from HSR images. From a rural management perspective, this work describes a scheme for rapid identification of rural settlements in a large region by using HSR images. The classification method presented here could be extended to the identification of rural settlements in a larger area, and the results can be used as a guide for on-site verification or enforcement in cadastral inventory.

In future works, further improvements could be made by integrating multi-temporal HSR images and multi-modal data, so that the dynamics of rural settlements can be characterized.

## Figures and Tables

**Table 1:** The number of testing samples.

	LDS	HDS	Backgrounds	Sum
Point-based testing samples	6125	2616	2887	11,628
Polygon-based testing samples	1831	438	/	2269

**Table 2:** Confusion matrix of point test set.

		Predicted Class			
		LDS	HDS	Backgrounds	Sum
Ground truth	LDS	5997	3	125	6125
	HDS	4	2551	61	2616
	Backgrounds	4	0	2883	2887
	Sum	6005	2554	3069	11,628
	UA	99.87%	99.88%	93.94%	
	PA	97.91%	97.52%	99.86%	
	OA	98.31%			
	Kappa	0.9724			

**Table 3:** Confusion matrix of polygon test set (m^2^).

		Predicted Class			
		LDS	HDS	Backgrounds	Sum
Ground truth	LDS	720,551	9228	118,198	847,977
	HDS	2673	349,060	60,476	412,209
	Backgrounds	95,539	51,323	24,231,862	24,378,724
	Sum	818,763	409,611	24,410,536	25,638,910
	UA	88.00%	85.22%	99.27%	
	PA	84.97%	84.68%	99.40%	
	OA	98.68%			
	Kappa	0.8591			

**Table 4:** Model comparisons with baseline, where values in bold are the best.

	OA	UA		PA		Kappa
		LDS	HDS	LDS	HDS	
Res50Seg (Baseline)	98.36%	82.50%	80.45%	83.30%	67.75%	0.8329
+Dilation	98.39%	84.25%	78.76%	80.53%	76.90%	0.8363
+Dilation+Multiscale	98.53%	87.24%	84.88%	81.90%	83.19%	0.8513
+Dilation+Multiscale+SE (Ours)	**98.68%**	**88.00%**	**85.22%**	**84.97%**	**84.68%**	**0.8591**

**Table 5:** Accuracy assessment of different methods, where values in bold are the best.

Method	OA	UA		PA		Kappa
		LDS	HDS	LDS	HDS	
OBIA	97.54%	75.24%	71.44%	72.24%	79.95%	0.7397
FCN	97.46%	73.11%	75.44%	70.28%	55.46%	0.7205
UNet	98.39%	84.58%	77.08%	80.32%	66.45%	0.8245
SegNet	98.37%	84.06%	78.51%	80.20%	68.79%	0.8232
DeeplabV3+	98.69%	87.92%	83.43%	**85.51%**	82.93%	0.8520
Ours	**98.68%**	**88.00%**	**85.22%**	84.97%	**84.68%**	**0.8591**

**Table 6:** The efficiency of different methods.

Method	Parameters	Training Time	Inference Time
OBIA		~0.5 h	~10 m
FCN	12.38 million	~3.1 h	0 m 17 s
UNet	33.40 million	~11.8 h	0 m 39 s
SegNet	29.44 million	~ 8.2 h	0 m 31 s
DeeplabV3+	39.76 million	~12.9 h	0 m 32 s
Ours	28.04 million	~5.8 h	0 m 25 s

## Supplementary Materials

The following are available online at https://www.mdpi.com/1424-8220/20/21/6062/s1, Table S1: Specification of our network architecture.
