# Supplementary material for: Mapping and Discriminating Rural Settlements Using Gaofen-2 Images and a Fully Convolutional Network
Source: Sensors (Basel). 2020 Oct 25;20(21):6062. doi: 10.3390/s20216062 (PMC7662595; doi:10.3390/s20216062)
Supplement: Supplementary file 1 [file sensors-20-06062-s001.pdf]

Table S1: Specification of our network architecture

Name	Kernel	Str.	Input	OutDim
Img	**Input image**			$H \times W \times 4$
Base	$7 \times 7, 64$	2	Img	$\frac{1}{2}H \times \frac{1}{2}W \times 64$
Pool0	3×3	2	Base	$\frac{1}{4}H \times \frac{1}{4}W \times 64$
Res1	$\begin{bmatrix} 1 \times 1, 64 \\ 3 \times 3, 64 \\ 1 \times 1, 256 \end{bmatrix} \times 3$	1	Pool0	$\frac{1}{4}H \times \frac{1}{4}W \times 256$
Res2	$\begin{bmatrix} 1 \times 1, 128 \\ 3 \times 3, 128 \\ 1 \times 1, 512 \end{bmatrix} \times 3$	2	Res1	$\frac{1}{8}H \times \frac{1}{8}W \times 512$
Res3	$\begin{bmatrix} 1 \times 1, 256 \\ 3 \times 3, 256 \\ 1 \times 1, 1024 \end{bmatrix} \times 3$, dila.=2	1	Res2	$\frac{1}{8}H \times \frac{1}{8}W \times 1024$
Res4	$\begin{bmatrix} 1 \times 1, 512 \\ 3 \times 3, 512 \\ 1 \times 1, 2048 \end{bmatrix} \times 3$, dila.=5	1	Res3	$\frac{1}{8}H \times \frac{1}{8}W \times 2048$
Conv4	$1 \times 1, 256$	1	Res4	$\frac{1}{8}H \times \frac{1}{8}W \times 256$
Conv3	$1 \times 1, 256$	1	Res3	$\frac{1}{8}H \times \frac{1}{8}W \times 256$
Conv2	$1 \times 1, 256$	1	Res2	$\frac{1}{8}H \times \frac{1}{8}W \times 256$
**Concat**				
Feat5_0		1	Conv4<br>Conv3<br>Conv2	$\frac{1}{8}H \times \frac{1}{8}W \times 768$
Feat5_1	$3 \times 3, 256$	1	Feat5_0	$\frac{1}{8}H \times \frac{1}{8}W \times 256$
Feat5_2	$1 \times 1, 256$	1	Feat5_1	$\frac{1}{8}H \times \frac{1}{8}W \times 256$
**SE block**				
Feat5_3	Please refer to [1] for details		Feat5_2	$\frac{1}{8}H \times \frac{1}{8}W \times 256$
Feat5_4	$3 \times 3, 256$	1	Feat5_3	$\frac{1}{8}H \times \frac{1}{8}W \times 256$
Res1_1	$1 \times 1, 48$	1	Res1	$\frac{1}{4}H \times \frac{1}{4}W \times 48$
**Concat**				
Merge_0	Bilinear upsampling 	1	Feat5_4<br>Res1_1	$\frac{1}{4}H \times \frac{1}{4}W \times 304$
Output	$3 \times 3, 3$	1	Merge_0	$H \times W \times 3$

	Bilinear upsampling				

1. Hu, J.; Shen, L.; Albanie, S.; Sun, G.; Wu, E. Squeeze-and-Excitation Networks. *IEEE Transactions on Pattern Analysis and Machine Intelligence* **2019**, 1–1, doi:10.1109/TPAMI.2019.2913372.
